# A Flow Cytometry-Based Serological Assay to Detect Visceral Leishmaniasis in HIV-Infected Patients

**DOI:** 10.3389/fmed.2021.553280

**Published:** 2021-04-30

**Authors:** Elis D. da Silva, Beatriz C. de Oliveira, Allana M. de S. Pereira, Diego L. Guedes, Osvaldo P. de Melo Neto, Carlos H. N. Costa, Zulma M. de Medeiros, Valéria R. A. Pereira

**Affiliations:** ^1^Aggeu Magalhães Institute, Oswaldo Cruz Foundation, Recife, Brazil; ^2^Laboratory of Leishmaniasis, Natan Portella Institute of Tropical Medicine, Teresina, Brazil

**Keywords:** visceral leishmaniasis, HIV infection, aids, diagnosis, flow cytometry

## Abstract

Visceral Leishmaniasis (VL) is a severe parasitic disease that has emerged as an important opportunistic condition in HIV-infected patients and whose control is impaired by inaccurate identification. This is mainly due to the serological tests used for VL having a reduced performance in cases of VL-HIV coinfection due to a low humoral response. In this situation, however, a positive test has even greater diagnostic value when combined with the clinical status. This study aimed to evaluate the application and performance of flow cytometry to detect anti-*Leishmania infantum* antibodies in HIV-infected patients. Sera from VL/HIV coinfected patients, characterized using “gold standard” techniques, were compared with sera from healthy controls plus sera from HIV-infected individuals. The flow cytometry results were expressed as levels of IgG reactivity, based on the percentage of positive fluorescent parasites (PPFP). A ROC curve analysis of a serum titration indicated a PPFP of 1.26% as being the cutoff point to segregate positive and negative results. At the 1:2,048 dilution, with 89% sensitivity and 83% specificity, flow cytometry showed greater sensitivity in relation to the serological tests evaluated. Futhermore, flow cytometry was the only assay that positively identified all VL-HIV patients with quantified HIV load. Together, these findings suggest that flow cytometry may be used as an alternative serological approach for VL identification and as a tool to characterize the humoral response against *Leishmania infantum* in HIV-infected patients.

## Introduction

Visceral Leishmaniasis (VL) is a potentially fatal disease that has emerged as an important opportunistic condition in HIV infected patients, resulting in a substantial number of VL–HIV coinfection cases which have been reported from 35 countries. The coinfection generates an impact in the immunopathogenesis, clinical manifestation, therapeutic response and diagnosis of both diseases (1). Case definition of HIV-VL requires confirmation of HIV infection by serological tests and positive results for VL diagnosis based on parasitological (bone marrow aspirate), serological or molecular methods, in addition to clinical symptoms. The microscopic examination or isolation of the parasite, the protozoan *Leishmania infantum*, is considered the gold standard for laboratorial confirmation of VL. Although this technique has high specificity, its use in clinical laboratories has some limitations, mainly due to the low sensitivity levels. The procedures involved are also invasive, time-consuming and require experienced personnel (2). Furthermore, due to the immunodepressed status of HIV-infected individuals, the parasites may not be found in the bone marrow, but rather in less common sites such as the oral mucosa, skin, stomach, colon and lungs (3–5). Serological approaches which detect specific antibodies against *L. infantum* constitute a valuable alternative as an early, rapid, and user-friendly diagnostic test. In the VL-HIV coinfection, however, the conventional VL serological assays, which includes indirect immunofluorescence test and the rK39 rapid test, are not considered accurate due to the low antibody production in these individuals (6–8).

The development of an effective VL diagnosis for the VL-HIV coinfections represents still a relevant challenge since it needs to be precise in order to reduce the lethality and mortality of afflicted individuals. Considering the limitations of the available diagnostic techniques, alternative methodologies have been employed (9, 10). One of them is flow cytometry, a technique that has been seen to be useful for a diversity of diagnostic applications, such as immunodeficiency disorders and cancer (11, 12). In addition, it can also be applied to parasitic diseases, such as Chagas Disease and leishmaniasis (13, 14). This technique has several advantages for immunoassays, such as high throughput capacity, possibility of analyte quantification, reduced sample volume, high reproducibility and sensitivity (14, 15). More importantly, it allows the development of multiplex studies using recombinant antigens, and it can be used as a monitoring tool for cured patients, allowing a more sensitive detection of anti-*Leishmania* antibodies (16–18). Therefore, the aim of this study was to evaluate the performance and to verify the possible application of an alternative diagnostic method using flow cytometry to detect anti-*L. infantum* antibodies in HIV-infected patients.

## Methods

### Serum Samples and Study Population

The study population was defined by the convenience of the sample size from two states from Northeastern Brazil (Pernambuco and Piaui). The sera used were from 18 VL-HIV coinfected (diagnosed by *Leishmania* positive bone marrow aspirate and rapid HIV test) and 18 VL negative-HIV positive patients as well as 18 healthy control individuals, with VL negative sera confirmed using conventional serological tests (rK39 rapid test and DAT). For the VL-HIV coinfected group, eight patients (five from Pernambuco and three from Piaui) had been more thoroughly investigated prior to this study during their clinical evaluation, with more detailed immunological records available (CD4 T cell count and viral load). All serum samples were collected in vacutainer tubes (BD Biosciences), processed by centrifugation (1,000 g, 10 min, room temperature), inactivated by heating (30 min at 56°C) and centrifuged at 4°C, 1,000 g for 5 min. After centrifugation, the supernatants were aliquoted and kept at −20°C until further use.

This study was approved by the Ethics Committees from the Federal University of Piauí (0116/2005) and from the Aggeu Magalhães Institute, Oswaldo Cruz Foundation (CAEE 51603115.7.0000.5190).

### Conventional Tests for VL Diagnosis

Bone marrow (1 mL) aspirates were obtained for *Leishmania* detection and used to prepare smears by slide apposition. The slides were stained with a panoptic staining kit (Ranylab, Barbacena, Brazil) and were evaluated under a light microscope (100 × objective). At least three bone marrow smears were evaluated for each patient and the process was performed according to Da Silva et al. (19). Rapid tests based on rK39 (IT LEISH) were purchased from Bio Rad Laboratories (Marnes-la-Coquette, France) and performed according to the manufacturer's instructions. The DAT was carried out according to the manufacturer's instructions (Royal Tropical Institute, Amsterdam, NL), with sera having dilution titers of 1:6,400 considered positive, as defined by El Harith et al. (20).

### In-house Immunofluorescence Antibody Test

The IFAT test was performed with an in-house protocol, where 20 μl of a *L. infantum* promastigote antigenic suspension were applied to the delimited region of IFAT slides (PERFECTLAB, São Paulo, Brazil) and kept for 2 h at 37°C. The slides were then coated with 10 μl of the patients' serum, in titers ranging from 1:20 to 1:320, diluted in PBS, pH 7.2. Two control sera (positive and negative) were incubated in a humid chamber for 30 min at 37°C. After incubation, the slides were washed three times through immersion in PBS, in intervals of 10 min. Anti-human IgG conjugated to fluorescein isothiocyanate-FITC (Sigma Chemical Corp., St. Louis, MO) prepared in Evans blue (40 mg) in PBS (previously diluted at 1:10 ratio in the same buffer) was added to the slides in a 1:50 dilution, and incubated under the same conditions as mentioned before. The slides were then washed three times for 10 min in PBS and left at room temperature. The assembly was made with buffered glycerin pH 8.5 and the slides then observed under a fluorescence microscope, with a 100 × objective. Sera were considered positive from the dilution 1:40.

### ELISA

The ELISA test was performed as described by Oliveira et al. (21), using 600 ng per well of crude *L. infantum* antigen assayed with the various sera diluted 1:900, followed by incubation with the peroxidase-conjugated anti-IgG (Calbiochem, EMD Millipore, Billerica, MA) diluted 1:2,000. After enzymatic detection with o-phenylenediamine (OPD) and H_2_O_2_, the reaction was quenched by adding 2M H_2_SO_4_ (50 μl/well) and the plates read at 490 nm (Spectra Max 190, Molecular Devices, Sunnyvale, USA or MRX II, Dynex Technologies, Chantilly, USA). Positive and negative controls were added to each 96-well plate to standardize the readings and variations. The cutoff point between non-reagent and reagent samples was calculated as the mean of the negative controls plus two standard deviations.

### Flow Cytometry

The flow cytometry assay was performed as originally described by Rocha et al. (22). Cultured *L. infantum* promastigotes (strain MHOM/BR/70/BH46) were harvested and washed three times in ice-cold PBS supplemented with 10% fetal bovine serum (FBS), prior to resuspension in 1% paraformaldehyde and incubation overnight. Following by a new wash and resuspension in PBS+ 10% FBS, the parasite suspension was incubated in 96-well, U-bottom plates (2.5 × 10^5^/well) at 37°C for 30 min in the presence of different serum dilutions (1:64–1:8,192), followed by two washes with PBS-10% FBS. The parasites were then incubated at 37°C for 30 min protected from the light and in the presence of anti-human IgG conjugated to fluorescein isothiocyanate-FITC (Sigma Chemical Corp., St. Louis, MO) diluted 1:200 in PBS– 10% FBS. After yet another wash, FITC labeled parasites were fixed with 200 μL of 1% paraformaldehyde and kept away from direct light for 30 min at 4°C until data acquisition on the flow cytometer (FACScalibur, Becton Dickinson), using the software “Cell Quest Pro,” with 20.000 events per sample. Promastigotes were identified based on their specific frontal (FSC) and side (SSC) light scattering properties. After FSC and SSC gain adjustments, the parasites assumed a characteristic distribution with these parameters. The relative FITC fluorescence intensity of each event was analyzed with a single histogram representation. A delimitation was set on the FITC-conjugated internal control histogram and it was applied to all data analyses reported here in order to determine the percentage of positive fluorescent parasites (PPFP) for each sample (Supplementary Figure 1). The optimal serum dilution and PPFP cutoff point were then selected to gather the IgG reactivity data with the best performance indexes. The values obtained were plotted as the mean of the PPFP related to the inverse dilution of the evaluated sera. For each assay, in addition to the FITC-conjugated internal control, unlabeled controls in quadruplicates and negative (a pool of negative sera) and positive (a pool of positive sera) controls were included to validate the assay.

### Statistics

For each test, the sensitivity was determined as the fraction of the confirmed VL-HIV coinfected sera that were reagent, and the specificity was calculated as the fraction of non-reagent sera (Healthy controls and HIV mono–infected groups) that were identified to be truly test negative. Statistical analyses were performed using a two-by-two contingency table with exact binomial 95% CIs using the OpenEpi Software (Version 2.3.1, Centers for Disease Control, Atlanta, GA, USA). The degree of agreement was determined by the kappa index, using the Landis and Koch interpretation criteria. A kappa-value of 0.60–0.80 represents a substantial agreement beyond chance and a kappa-value of >0.80 represents almost perfect agreement beyond chance (23). The graphs were generated by the GraphPad Prism version 7.0 (GraphPad Prism Inc., San Diego, CA).

## Results

### Defining the Flow Cytometry Parameters for the Diagnosis of VL-HIV Coinfected Cases

**Figure 1:**
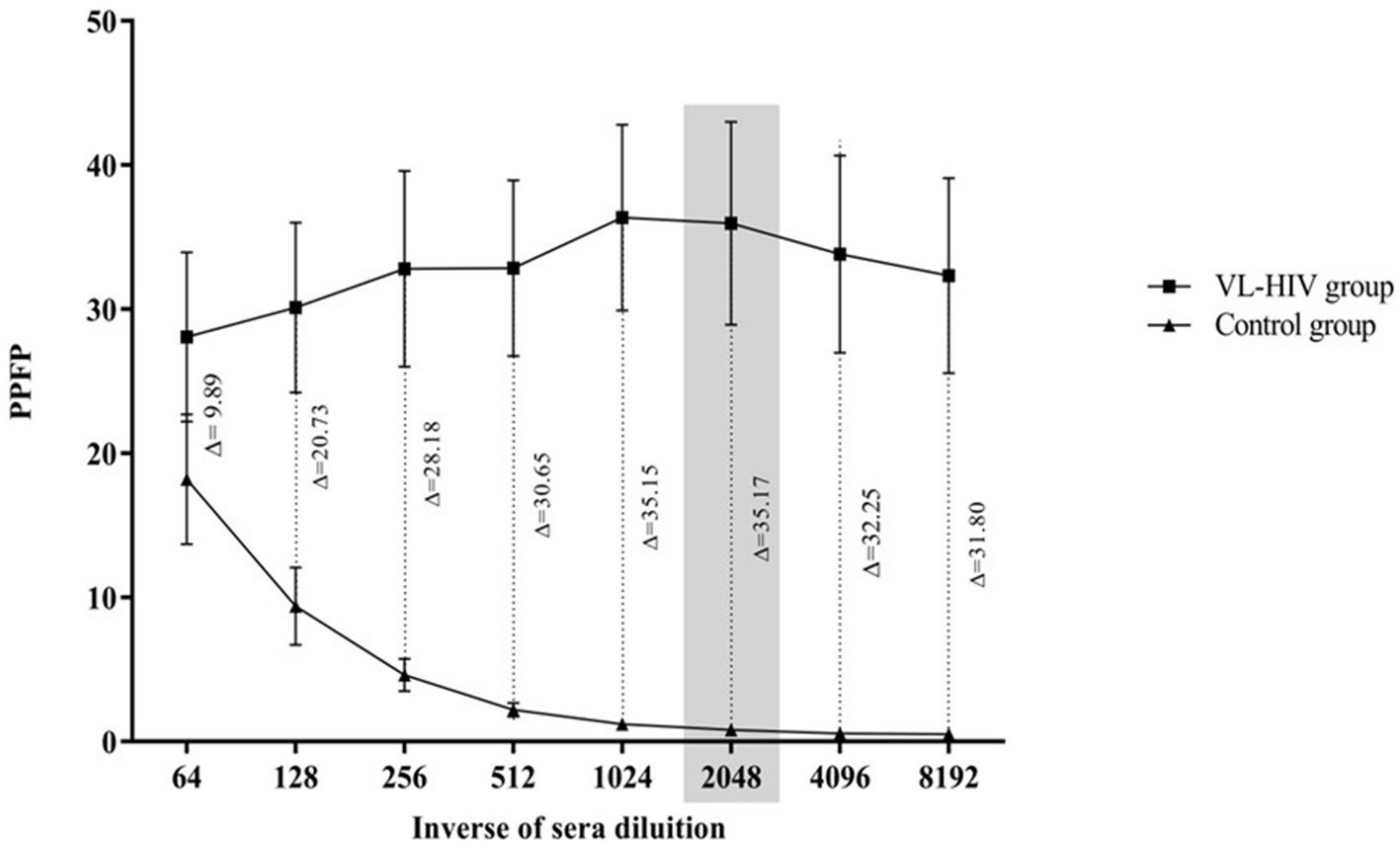
IgG antibody titration curve of anti-fixed *Leishmania infantum* promastigotes defected by flow cytometry with sera from the two stratified groups assayed here. (|) VL-HIV group, *n* = 18, and (▴) control group, *n* = 36. The gray rectangle corresponds to the titration of 1:2,048 which was the region of greatest separation between groups. PPFP, Percentage of Positive Fluorescent Parasites. Δ = difference seen for the reacitivity between groups.

To evaluate the use of flow cytometry serology to clearly differentiate between positive and negative VL samples from HIV co-infected individuals, a serum dilution curve was used to assess the IgG reactivity data from sera from VL-HIV coinfected patients in comparison with a VL-negative control group. The VL-HIV coinfected samples all consisted of true positive cases identified by a positive parasitological test for VL and positive HIV serology. The control group consisted of sera from healthy individuals, with no obvious signs and symptoms of any disease and living in the non-endemic regions for VL, as well as HIV mono-infected individuals, with positive serology for HIV and negative serology for VL. This negative VL serology was confirmed through three independent assays: an in-house Immunofluorescence Antibody Test (IFAT), a commercial Direct Agglutination Test (DAT) and ELISA using a crude *L. infantum* antigen preparation. Figure 1 shows the mean values of the percentage of positive fluorescent parasites (PPFP) from VL-HIV coinfection and control groups vs. a sera dilution curve ranging from 1:64 to 1:8,192. The difference between the reactivity of positive and negative samples (Δ) showed that the best performance in segregating these groups was at the dilution of 1:2,048. Thus, we used this dilution to better define the optimal PPFP value for VL diagnosis.

### Defining a Cutoff Point for the Diagnosis of VL-HIV Coinfected Cases

**Figure 2:**
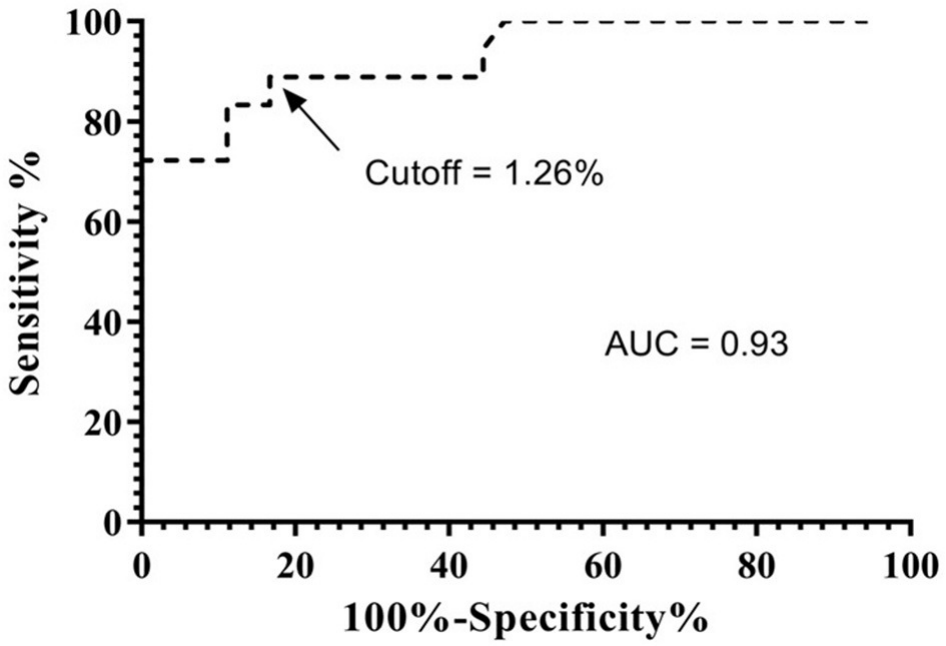
ROC curve analysis of the performance indexes, sensitivity, and specificity of flow cytometry. The ROC curve was applied to confirm the best cutoff point which was able to discriminate PPFP values from positive and negetive samples and to indicate the area under the curve (AUC = global accuracy). ROC curve of the samples at dilution 1:2,048 indicating a cutoff point of 1.26.

Next, we sought to define an ideal cutoff point for the flow-cytometry, which would be able to differentiate the IgG reactivity data with the best performance indexes. This was evaluated through a Receiver Operating Characteristic (ROC) curve, generated by plotting sensitivity on the y-axis and the complement of specificity (100—specificity) on the x-axis and thus able to discriminate negative from low positive and high positive PPFP results. The data analysis of the ROC curve demonstrated that the PPFP value of 1.26 was the most appropriated cutoff to distinguish negative (PPFP ≤ 1.26%) from positive (PPFP > 1.26%) results (Figure 2). The tests' global accuracy determined by the area under the ROC curve (AUC), which was calculated at 0.93 [95%, with a confidence interval (CI) between 0.85 and 1.0]. Using this approach, flow cytometry displayed 89% of sensitivity (CI 95% = 65–99%) and 83% of specificity (CI 95% = 67–94%). The mean PPFP values was 36% (CI 95% = 22–50%) for the VL-HIV coinfection group, 1.4% (CI 95% = 0.9–1.8%) for the healthy controls and 0.2% (CI 95% = 0.1–0.35%) for the mono-infected HIV group.

### Comparative Analysis of Flow Cytometry and Conventional Serological Tests for VL-HIV Diagnosis

Aiming to evaluate the global performance of flow cytometry, we used the same serum panel with serological tests conventionally used for the diagnosis of VL (DAT, rK39 rapid test, ELISA, and IFAT). Flow cytometry had the best values in terms of sensitivity and negative predictive value (89 and 94%, respectively), however, the other tests were more specific (100%) when compared to flow cytometry, which had a specificity of 83% (Table 1). When the different tests were individually compared to flow cytometry, we could identify a substantial agreement between DAT and ELISA tests (κ > 0.6; agreement > 80%) and a moderate agreement between rK39 rapid test and IFAT (κ <0.6; agreement <80).

**Table 1:** Values of sensitivity, specificity, positive and negative predictive values, and accuracy of the serological tests used for the diagnosis of VL-HIV coinfection (*N* = 54)^*^.

	**Flow cytometry**	**DAT**	**rK39 rapid test**	**ELISA**	**IFAT**
Sensitivity (95%CI)^a^	89% (67–97%)	83% (61–94%)	72% (49–87.5%)	72% (49–87.5%)	61% (39–80%)
Specificity (95%CI)	83% (68–92%)	100% (90–100%)	100% (90–100%)	100% (90–100%)	100% (90–100%)
PPV^b^ (95%CI)	73% (52–87%)	100% (80–100%)	100% (80–100%)	100% (80–100%)	100% (74–100%)
NPV^c^ (95%CI)	94% (80–98%)	92% (80–97%)	88% (74–95%)	88% (74–95%)	84% (70–92%)
Accuracy (95%CI)	85% (73–92%)	94% (85–98%)	91% (80–96%)	91% (80–96%)	87% (76–94%)

* *The samples included 18 VL-HIV coinfected patients, 18 VL negative-HIV positive patients and 18 healthy individuals (negative control)*.

a *CI, Confidence Interval*.

b *PPV, Positive Predictive Value*.

c *NPV, Negative Predictive Value*.

### Performance of Flow Cytometry and Standard Serological Tests in Relation to the Immunological Status of Patients Co-Infected With VL-HIV

Eight of the VL-HIV coinfected sera were derived from patients whose immune statuses had been evaluated and the HIV viral load quantified (Table 2). We observed that flow cytometry was able to positively identify all of these sera, including those with a more severe immunossupression, with CD4+ T cell counts below 200 cells/mm^3^, and even taking into account the very large variations in HIV viral load. Although the various conventional tests evaluated also gave positive results even in the patients with the most severe immunosuppressions, all the other tests had at least one negative result for the series of cases analyzed, with IFAT having the worst performance (three false negative results). However, no clear correlation between immunosuppression, viral load and positivity in the assays was observed for the any of these tests.

**Table 2:** Laboratorial findings of eight cases of the VL-HIV/AIDS coinfected group.

**Patient**	**Flow cytometry (%PPFP)**	**DAT (Titer)**	**rK39 rapid test**	**ELISA (Absorbance-490 nm)**	**IFAT (Titer)**	**T CD4+ (cells/mm^**3**^)**	**Viral load (copies/mL)**
1	Positive (1.37)	Positive (1:51,200)	Positive	Positive (0.64)	Negative	399	3,722
2	Positive (23.47)	Positive (1:24,600)	Positive	Positive (0.85)	Positive (1:160)	56	50,000
3	Positive (1.96)	Negative	Negative	Negative (0.02)	Negative	392	<50
4	Positive (37.91)	Positive (1:102,400)	Positive	Positive (3.5)	Positive (1:160)	2	<50
5	Positive (21.56)	Positive (1:51,200)	Positive	Positive (0.85)	Positive (1:160)	<50	54
6	Positive (59.76)	Positive (1:51,200)	Positive	Positive (0.65)	Positive (1:40)	157	45,795
7	Positive (4.89)	Positive (1:6,400)	Negative	Negative (0.04)	Negative	345	39,529
8	Positive (88.32)	Positive (1:51,200)	Positive	Positive (3.1)	Positive (1:320)	92	1,027

## Discussion

To our knowledge, this is the first study using the detection of antibodies anti-*L. infantum* by flow cytometry for VL diagnosis in HIV-infected individuals. Previous reports started by using this technique to evaluate IgG binding to live promastigotes to assay individuals with VL (24, 25) and with American tegumentary leishmaniasis (26). The used of fixed cells as an alternative was also investigated with both Chagas disease and tegumentary leishmaniasis (27, 28). In a previous study, we also directly investigated the use of fixed promastigotes to assess IgG binding by flow cytometry for the diagnosis of individuals with VL, reaching a sensitivity of 92–96% in these individuals (15). In the present study, we found a good, but not ideal, sensitivity, although in relation to the conventional tests used for comparison, the sensitivity of flow cytometry was greater. As described here, the technique is even more relevant for the diagnosis of VL in cases of VL-HIV co-infections.

The rk39 rapid test and IFAT are the serological tests most used for the diagnosis of VL, but they show the lowest sensitivity (<60%) in VL-HIV coinfected individuals (6). Therefore, particularly for this group of patients, the VL diagnosis is a great challenge. Our results showed higher sensitivity levels for these tests than previously reported, but with a performance still inferior to flow cytometry and DAT.

Indeed, among the serological tests conventionally used for the VL diagnosis in coinfected individuals, DAT stands out as having the highest sensitivities in multiple studies: 89% (29), 81% (6), 82.3–89.7% (30), 91.3% (31), 89.5% (32), and 90% (33). This performance was also corroborated by our study. Both DAT and flow cytometry use serial dilutions that allow the identification of antibodies in low serum concentrations, even in immunosuppressive conditions (CD4+ T cells <200 cells/mm^3^). Nevertheless, flow cytometer uses photomultiplier detectors and its quantitative assessment excludes the operator subjectivity which exists in DAT. In this context, flow cytometry shows the potential to be an alternative serological method for VL detection in HIV-infected patients, since a positive test, even at low titers, has diagnostic value when combined with the clinical case definition.

All serological tests, except for flow cytometry, had 100% specificity. This may have been overestimated in our study, since we used the rK39 rapid test and DAT for the original screening for the group of VL negative-HIV positive samples. In previous studies, specificities varying from 83.3 to 90% for DAT and 97.4 to 100% for rapid tests have been observed in VL-HIV coinfections (6, 31, 32). Ideally, it would be best to evaluate different control groups, such as individuals from endemic regions and with other confirmed pathologies, to have a more reliable specificity value. Despite the good sensitivity of our flow cytometry data, further investigations are needed in order to reduce the high incidence of false positive results seen here among healthy controls from non-endemic regions (Supplementary Figure 2). It is particularly important to investigate VL-related diseases that are co-endemic, since cross-reactivity with other trypanosomatid infections still represents an important issue regarding the applicability of flow cytometry (24, 34).

So far, it has been a challenge to find a more practical and safer antigen preparation which would allow greater sensitivity and specificity levels with low cross-reactivity. Improvements which include the use of fixed parasites and solutions that are able to preserve their morphology, such as formaldehyde, were strategies developed to facilitate the use of these parasites and to enable the development of diagnostic kits (35, 36). It is also noteworthy that the development of algorithms which allow the elimination of cross-reactivity are important for the differential diagnosis of trypanosomatids (14), but the use of molecularly defined antigens seems to be the best option capable of addressing this limitation (16).

With the emergence of monoclonal antibodies and flow cytometry, it was possible to clarify the role of CD4+ T cells in HIV-AIDS. Indeed, CD4 quantitation is currently one of the most widespread tests performed in diagnostic centers for the prognosis and evaluation of anti-retroviral treatments in HIV-infected individuals (37). In our study, the immunological data were collected retrospectively from medical records, limiting the complete evaluation of all patients. Nevertheless, for those with the data available, flow cytometry was able to detect anti-*L. infantum* antibodies even in cases with low CD4+ T cell counts. It can thus be an additional tool to improve the evaluation of individuals in endemic regions for VL with lower CD4+ T counts. **In this context**, it would be interesting to take advantage of the operational and technical settings that flow cytometers use to quantify CD4 and apply them also for the detection of anti-*Leishmania* antibodies in countries which are endemic for VL, such as Brazil. This would imply adding an algorithm for the VL diagnosis in people with HIV from endemic areas, enabling a more sensitive diagnosis in cases with a prior negative VL result based on techniques such as DAT and rapid tests. As observed in a study carried out in Ethiopia, the need for a different algorithm for this population is evident due to the substantial reduction in the sensitivity of conventional techniques in HIV-infected individuals (38).

In conclusion, although it is a preliminary assessment, our results emphasize that flow cytometry can contribute to the correct identification of cases, especially in cases of immunosuppression, being a useful tool to characterize the humoral response to *Leishmania* in HIV-infected patients. Therefore, we encourage the evaluation of this technique in a larger number of samples and in other regions, such as those affected by *Leishmania donovani*.

## Data Availability Statement

The raw data supporting the conclusions of this article will be made available by the authors, without undue reservation.

## Ethics Statement

The studies involving human participants were reviewed and approved by Aggeu Magalhães Institute, Oswaldo Cruz Foundation, Recife, Pernambuco, Brazil. The patients/participants provided their written informed consent to participate in this study.

## Author Contributions

ES, ZM, and VP conceived and designed the study. ES, BO, and AP drafted the manuscript. ES and DG analyzed the data. ES, BO, AP, DG, OM, CC, ZM, and VP critically revised the manuscript. All authors read and approved the final manuscript.

## Conflict of Interest

The authors declare that the research was conducted in the absence of any commercial or financial relationships that could be construed as a potential conflict of interest.
