# Supplementary material for: A Flow Cytometry-Based Serological Assay to Detect Visceral Leishmaniasis in HIV-Infected Patients
Source: Front Med (Lausanne). 2021 Apr 30;8:553280. doi: 10.3389/fmed.2021.553280 (PMC8119745; doi:10.3389/fmed.2021.553280)
Supplement: Supplementary file 1 [file Data_Sheet_1.PDF]

Supplemental Figure 1

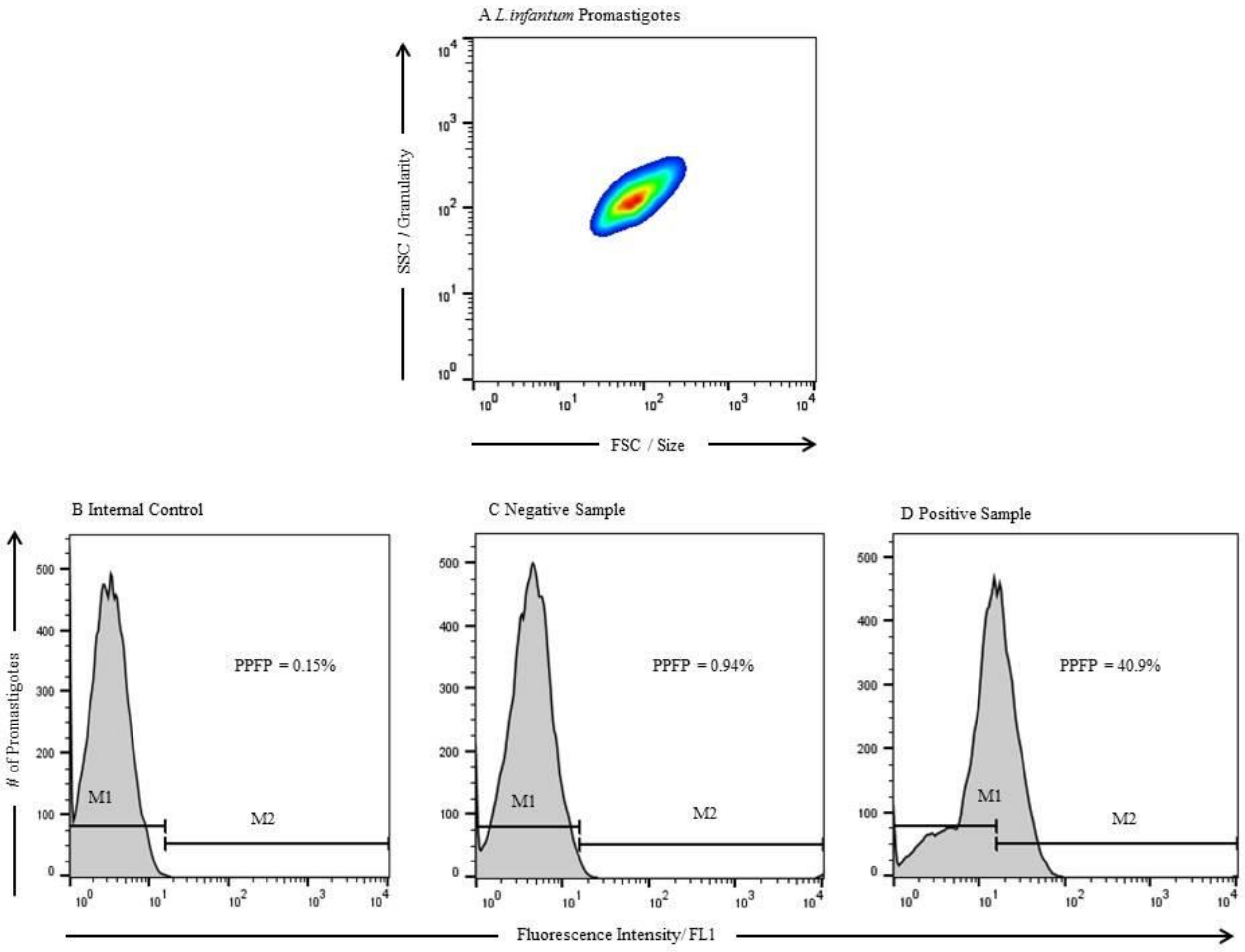

Fig 1. Representative flow cytometry serology charts used to analyze the anti-fixed *L. infantum* promastigotes IgG reactivity in human sera samples. Promastigotes were selected based on their FSC (size) versus SSC (granularity) on flow cytometry dot plots (A). The relative fluorescence intensity/FL1 was quantified in the absence of human sera but in the presence of the FITC-conjugated anti-human IgG (internal control). This condition leads to the establishing of a maximum value of reactivity and determination of the marker M1 (negative region) and M2 (positive region) (B). The reactivity of IgG is provided in histograms for each serum samples as PPFP, which represents the frequency of parasite shift toward higher fluorescence intensity, across the M2. This marker was maintained to determine the reactivity in all data analyses performed in serum sample from negative sample for VL (C) and positive sample for VL (D).

Supplemental Figure 2

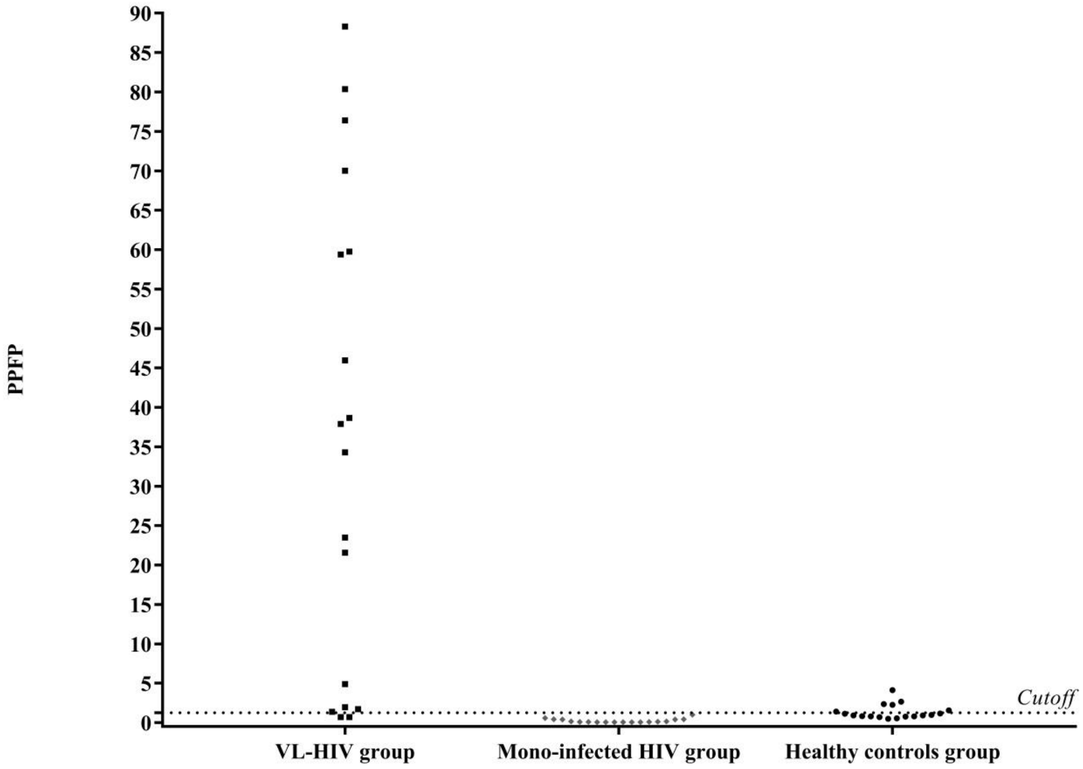

Fig 2. Anti-*L. infantum* IgG distribution. VL-HIV group (■) n = 18, mono-infected group (■) n =18 and Healthy control group (●) n = 18. All groups are at the 1:2048 dilution and the samples which are above the cutoff of 1.26% PPFP (Percentage of Positive Fluorescent Parasites) are considered positive.
